# Herbarium Specimens Reveal Long‐Term Decline in Pollination Services Since the 1970s

**DOI:** 10.1111/gcb.70793

**Published:** 2026-03-24

**Authors:** Bofeng Song, Heidi Zimmer, Mark Clements, Demetra Rakosy, Tiffany M. Knight, Joanne M. Bennett

**Affiliations:** ^1^ Fenner School of Environment & Society The Australian National University Canberra Australian Capital Territory Australia; ^2^ School of Biological Sciences The University of Western Australia Perth Western Australia Australia; ^3^ Centre for Australian National Biodiversity Research (Joint Venture Between Parks Australia and CSIRO) Canberra Australian Capital Territory Australia; ^4^ Thünen‐Institute of Biodiversity Braunschweig Germany; ^5^ German Centre for Integrative Biodiversity Research (iDiv) Leipzig Germany; ^6^ Department of Community Ecology Helmholtz Centre for Environmental Research—UFZ Leipzig Germany; ^7^ Department of Science and Conservation National Tropical Botanical Garden Kalāheo Hawaii USA; ^8^ Institute of Biology Martin Luther University Halle‐Wittenberg Halle Germany; ^9^ Gubali Institute for Agriculture, Water, and Environment Charles Sturt University Albury New South Wales Australia

**Keywords:** food deception, herbarium, Orchidaceae, pollination, sexual deception

## Abstract

Anthropogenic change has resulted in pollinator declines and altered plant–pollinator interactions. This may alter pollination services, reducing the reproductive success of plants. Yet few datasets allow us to track changes in pollination services over time. Herbaria provide a unique opportunity to assess pollination services across broad spatial and temporal scales enabling the examination of associated spatiotemporal anthropogenic drivers of change. We quantified changes in pollination services to the orchid genus *Caladenia* over the past century, a period of rapid land‐use intensification and climate change in Australia. Examining 10,494 *Caladenia* flowers preserved at the Australian National Herbarium showed a reduction in pollination services totaling > 60% over the whole study period, with rapid declines occurring post 1970. Declines in pollination services occurred across species pollinated by different taxa and with varying threat status. Sexually deceptive species showed more pronounced declines in pollination services than food‐deceptive species, whereas no decline was detected in the self‐compatible species. Land‐use intensity and rising temperatures were significant predictors of changes in pollination service. Our findings provide rare evidence of declines in pollination services and demonstrate the value of herbarium collections in understanding global change.

## Graphical abstract

Using over 10,000 *Caladenia* flowers from herbarium specimens, we reveal a nearly two‐thirds decline in pollination services since the 1970s, which is correlated with changes in climate.

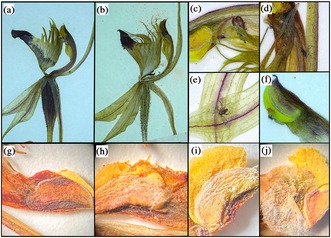

## 1 Introduction

Pollination is vital to the reproduction of ~80% of flowering plants (Rodger et al. 2021). Global evidence suggests that pollinators may be particularly vulnerable to human disturbance, raising concerns that we may be facing a “pollination crisis” where plant reproduction fails (Potts et al. 2016). There is growing evidence that land‐use change and high‐intensity farming practices characterized by reduced floral diversity, fragmented habitats and heavy reliance on synthetic inputs such as pesticides are having detrimental effects on pollinators (Goulson 2013). Indeed, on a global scale, plant reproduction has been shown to be more pollen‐limited in areas with high‐intensity land use (Bennett et al. 2020). Anthropogenic climate change is also likely to disrupt pollination services as rising temperatures are shifting species phenologies and ranges, placing pollinators out of sync with the timing and location of peak flowering for many plant species (Settele et al. 2016). Similarly, altered rainfall timing can shift flowering phenology (Van Dyke and Kraft 2025) and changes in rainfall amount can reduce floral display and nectar rewards, which diminishes pollinator attraction (Kuppler and Kotowska 2021). However, most evidence of disrupted pollinator services is found in studies that compare plant populations or across relatively short time periods in well‐studied regions. Direct evidence of declines in pollination services across longer time periods, relevant for understanding changes in land use and climate, is rare (Rakosy et al. 2023).

Herbaria hold collections spanning decades or centuries, which can be used to document spatial and temporal changes in pollination services (Rakosy et al. 2023). Data on rates of pollen transfer and ovary fertilization across multiple species and spatial scales has so far been rarely assessed (Rakosy et al. 2023). The family Orchidaceae is well‐represented within herbaria worldwide (Gaskett and Gallagher 2018; Molnár et al. 2012; Pauw and Hawkins 2011). Orchids, with a few exceptions, package pollen grains into pollinia that can be released through pollinator contact. The removal of the pollinium leaves an empty clinandrium (i.e., the part of the orchid column which houses the pollinium) which means pollen collection via a vector can be observed even in dried specimens. For example, historical pollinarium removal rates were assessed in *Pterygodium catholicum*, collected on Signal Hill, South Africa, from the late 19th century to 1950, revealing a decline over time (Pauw and Hawkins 2011). Similarly, a European study successfully used *Ophrys sphegodes* herbarium specimens to reconstruct long‐term phenological shifts driven by climate warming, further demonstrating that historical collections can reliably capture long‐term ecological trends in orchids (Robbirt et al. 2011).

Orchidaceae encapsulates extraordinary floral diversity, corresponding to their diverse and often highly specialized pollination systems (Cozzolino and Widmer 2005), as well as broader drivers of diversification such as highly heterogeneous habitats and complex evolutionary processes (Ackerman et al. 2023). Pollinator attraction relies on rewards in 54% of orchid species, and deceit in 46% of species, with orchids generally forming highly specialized relationships with individual pollination species (Ackerman et al. 2023). Sexual deception is common among orchid species and involves the production of sex pheromones and/or engaging in physical mimicry to attract specific pollinators (Schiestl et al. 2003; Peakall 2023). Rewarding species typically achieve higher fruit‐set rates (Trapnell and Hamrick 2006), whereas among deceptive orchids, sexually deceptive taxa generally attain higher pollination efficiency than food‐deceptive species (Scopece et al. 2010). Self‐pollinated species achieve the highest rates of pollination (Brundrett 2019). Pollination rates can also be affected by population density (which is often related to threatened species status), where smaller populations of orchids with specific pollinators often achieve higher pollination rates due to lower among‐plant competition for pollinators (Brundrett 2019). Hymenoptera is the predominant pollinator order in Orchidaceae, wasps (Thynnidae) and bees (Apidae) are key pollinators (Ackerman et al. 2023), with the former common in food‐ and sex‐deception, and the latter in food deception.

The Australian orchid genus *Caladenia* is relatively well represented across space and time in herbaria. There are around 288 species of *Caladenia* (Jones 2021), including 137 nationally threatened species (DCCEEW 2025), more than any other orchid genus in Australia. The genus encapsulates diverse pollination strategies, including self‐compatibility, food and sexual deception, and strategies intermediate among these (e.g., attracting pollinators using sexual deception but also providing tiny amounts of sugar on the labellum) (Phillips et al. 2023). Pollination success in *Caladenia* varies widely across pollination strategies. Sexually deceptive species generally exhibit very low pollination rates, averageing 14% ± 3% (Reiter et al. 2017, 2023; Phillips et al. 2009; Peakall and Beattie 1996). In contrast, food‐deceptive *Caladenia* species tend to achieve substantially higher pollination success, with reported rates of roughly 20%–40% (Phillips et al. 2009, 2020). Using specimens at the Australian National Herbarium (ANH) focusing on the 1920s to today, we assessed long‐term changes in pollination services to *Caladenia* species. We compared this with potential drivers of change, including climate (deviations in temperature, annual rainfall, and seasonal rainfall) and human land‐use intensity/footprint and whether observed changes were modulated by pollination strategy, pollinator taxonomic groups or threat status.

## 2 Methods

**FIGURE 1:**
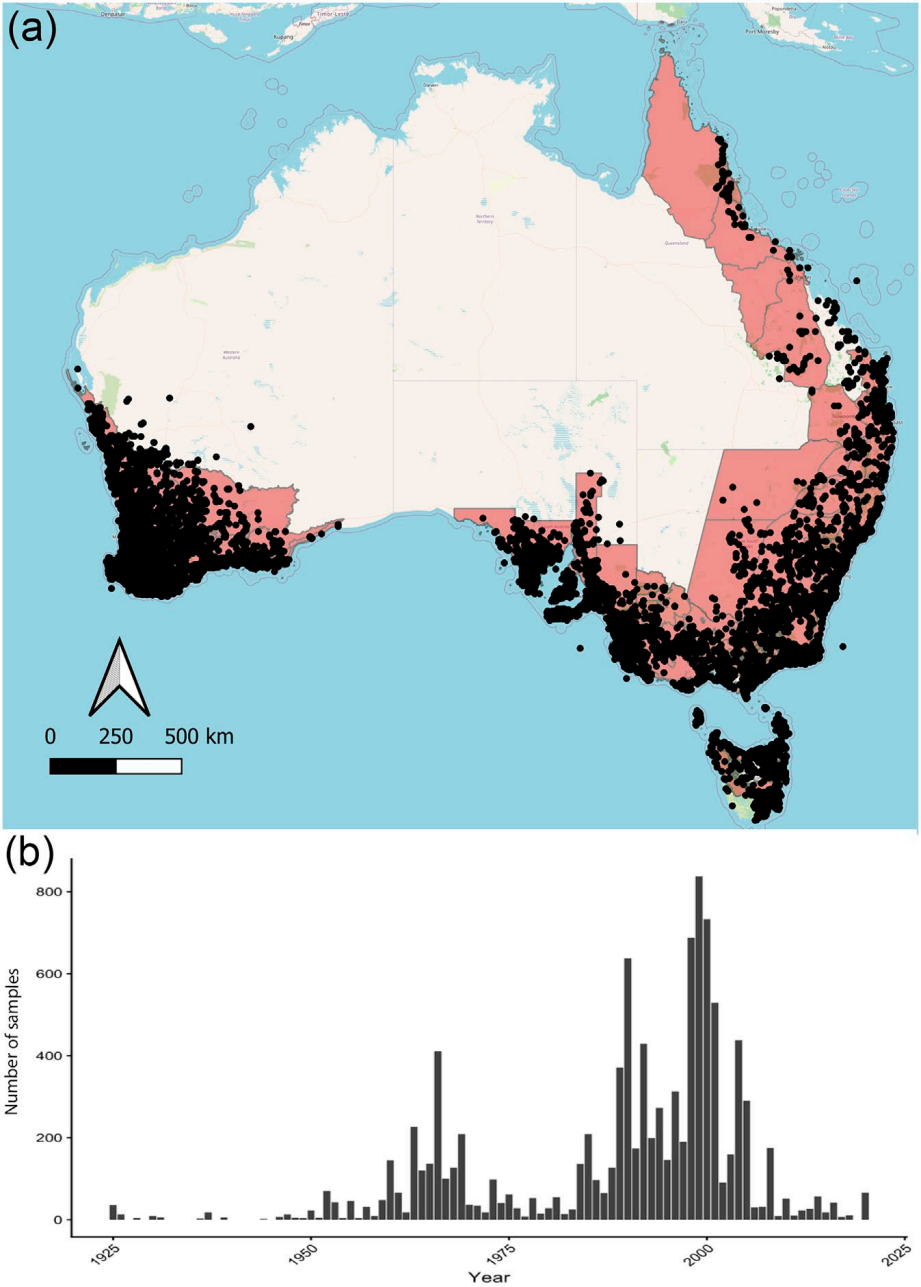
(a) Australian botanical districts represented in the dataset (red polygons) and the distribution of *Caladenia* occurrence records in Australia (black dots; preserved specimens only; ALA 2026). Basemap: OpenStreetMap. Map created using QGIS Association (2026). (b) Temporal distribution of specimens examined between 1925 and 2020, indicating the number of specimens assessed per year. Variation in annual counts reflects historical patterns in herbarium collection effort. Map lines delineate study areas and do not necessarily depict accepted national boundaries.

We accessed 2125 herbarium specimen sheets and examined 10,494 *Caladenia* flowers from the collection at the ANH. The dataset for this study included a total of 25 species, with collection localities spanning the known distribution of Australian *Caladenia* (Figure 1). These species were selected because they were the most well represented in the herbarium (each had more than 10 sheets) and represent different pollination strategies and major pollinator groups.

### 2.1 Assessment of Pollination Services Success

**FIGURE 2:**
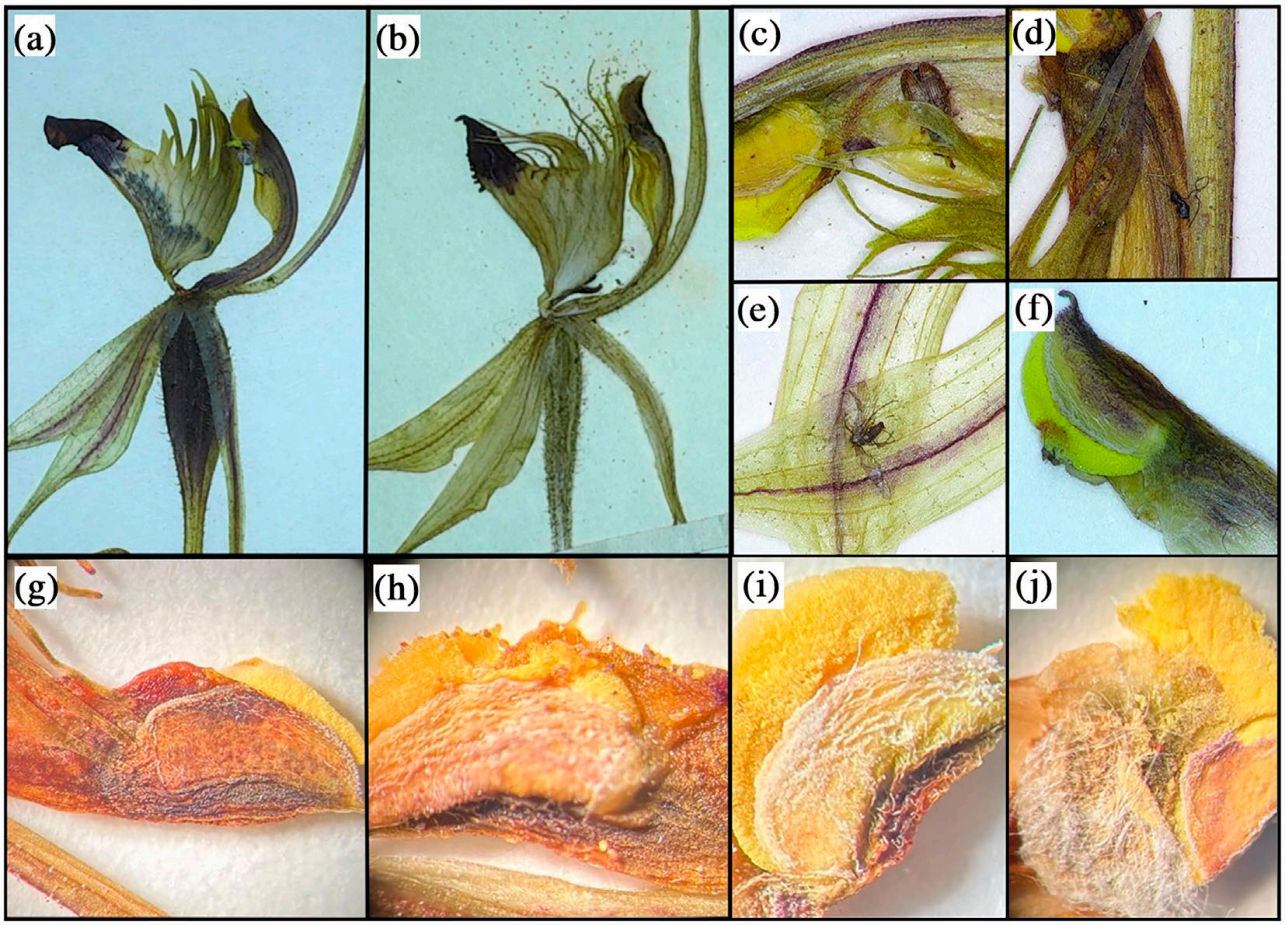
Herbarium‐based evidence of pollination services in *Caladenia* specimens. (a) A fertilized ovary, characterized by marked enlargement and darkening consistent with post‐pollination development. (b) An unfertilized ovary lacking morphological features associated with successful fertilization. (c–f) Evidence of insect visitation retained on floral structures, showing different forms of insect‐derived remains (e.g., legs or hairs) preserved on specimens. These observations indicate insect visitation but do not, on their own, confirm successful pollination and were therefore recorded as supporting evidence rather than included in quantitative analyses. (g–j) Evidence distinguishing alternative causes of pollinia condition and damage in *C. dilatata* . (g) Intact pollinia with no signs of insect visitation or human interference. (h) Pollinia damaged following insect visitation. (i) Pollinia damage attributable to collector error during specimen collection. (j) Human‐induced damage associated with specimen preparation, with visible white fibers representing remnants of thread used to secure floral structures.

For each herbarium sheet, flowers were labeled sequentially. Location information (latitude and longitude) and year of collection for each specimen was recorded from the specimen label. Only fully open flowers, accessible to potential insect visitors, were considered in this study. For open flowers we determined if pollinia were completely or partially absent from the clinandrium (where the anther is located) as evidence of pollination services for male reproduction (Figure 2). Complete pollinia removal was taken as evidence of pollination service. For partially removed pollinia, we determined whether the removal was likely insect‐related or caused by human handling. Pollinia breakage caused by insects is typically characterized by irregular or messy partial removal, consistent with the action of insect mouthparts or feet (Figure 2). Conversely, pollinia breakage caused by human handling was identified by distinct patterns such as tweezer punctures or straight cuts. In rare cases, damage could also occur during the mounting process such as when cotton threads used during specimen preparation adhered to the anther caps. These criteria, formalized through standardized decision trees, ensured that we could reliably distinguish between insect‐related and human‐related damage during our assessments (Figures S1 and S2).

To cross‐validate data on pollinia removal, we also collected information on pollination services to female reproductive parts. Specifically, we recorded if pollen was present on the stigmatic surface as supporting evidence of pollinator visitation, or if there was evidence of ovary fertilization assessed via a clear enlargement of the ovary width (Figure 2). We also recorded if insect parts were present on the stigma, but these data were not used in the analysis. All lines of evidence of pollination services were highly correlated. Most of the data were evidence for services to male reproduction *N* = 5861 (pollinium removal), with *N* = 954 records of pollination services to female reproduction (ovary fertilization and pollen on stigma).

### 2.2 Pollination Strategy and Taxonomic Specialization

For each species, we determined subgenera, pollination strategy, and major pollinators based on the literature (Table S1). The dataset comprised one self‐pollinating species ( *C. alata* ), 15 food‐deceptive species, and 9 sexually deceptive species. For the analysis of pollination strategies, species characterized by sexual deception and food deception were represented across the full dataset (1925–2020), whereas the self‐pollinating species only had sufficient data from 1980. In *Caladenia*, there are three main pollinator specializations: species that are primarily pollinated by either bees, wasps, or flies. Species for which pollinator specialization could not be determined or where uncertain (e.g., multiple pollinators were indicated but unknown) were excluded from analysis.

### 2.3 Threat Status

Species were denoted as threatened if they were listed regionally or nationally on the species profile and threat database under the Species Profile and Threats Database (DCCEEW 2025). Species not listed were treated as unlisted for the purposes of this analysis.

### 2.4 Data Analysis

To examine changes in pollination services to *Caladenia* across species grouped by different pollination strategies and major pollinator taxa, we used Generalized Additive Mixed Models (GAMM) fitted using the *mgcv* package (Wood 2001) in R (R Core Team 2025). To account for phylogenetic effects and species‐specific variability, nested random effects were included in the models. Specifically, the random effects were defined as subgenera (Subgenus) as a random intercept and species nested within subgenera (Species | Subgenus) to capture within‐group variation. The full dataset spanned from 1887 to 2020 and included 10,241 accessible flowers; however, due to the small sample size prior to 1925, the focus of the analysis was on the period from 1925 to 2020 (*n* = 10,178; Figure 1b, Figure S3). To evaluate differences in pollination services as a percentage across pollination strategies, a one‐way analysis of variance (ANOVA) was conducted. Post hoc Tukey HSD tests were performed to compare the mean differences between self‐pollinating, food‐deceptive, and sexually deceptive species. A Kruskal–Wallis test was performed to assess differences in the pollination services among pollinator groups (bees, wasps, and flies) during specific time periods. Post hoc comparisons were conducted to evaluate pairwise differences between groups.

We used the *segmented* package in R, to detect data‐driven significant shifts in trends and estimate breakpoints (Muggeo 2008). Segmented regression was applied to the overall dataset and independently to each pollination strategy (e.g., self‐pollination, food deception, sexual deception) and major pollinator specialization (e.g., bees, wasps, flies).

To explore potential correlations between changes in pollination services and changes in climate, we extracted national annual temperature and rainfall anomaly data from the Bureau of Meteorology (BOM 2024b, 2024a). Climate anomalies are measures of deviation from mean baseline conditions. Specimen records were linked to climate anomalies by collection year, and analyses were conducted using annual mean pollination services. For rainfall, in addition to the annual deviation in rainfall at the national scale, we classified records according to the broader seasonal rainfall zones of Australia; we classified each specimen record into summer or winter seasonal rainfall zones based on its botanical district, and extracted rainfall anomalies for the dominant periods of rainfall associated with those zones, summer or winter, respectively. We used Generalized Linear Model (GLM) with a Gaussian distribution and identity link, using pollination services as the dependent variable and environmental anomalies (temperature anomaly or rainfall anomaly, respectively) as the independent variable.

Land‐use intensity was taken from the human footprint index, which is a remotely sensed composite of the extent of human impact on the environment scaled from 0 to 1 (Venter et al. 2016). The index weights human pressures including the extent of built environment and infrastructure, human population density, light pollution and extent of crops and pastures based on their impact on the environment (Venter et al. 2016). The *raster* package in R was used to process and extract spatial raster data for the human footprint index, enabling precise alignment of spatial data with corresponding specimen locations (Hijmans et al. 2015). The *sf* package facilitated the handling and manipulation of spatial vector data, such as specimen collection points, ensuring accurate integration of geographic and environmental datasets (Pebesma 2018). A GLM with a Gaussian distribution and identity link function was applied to assess the effects of year, human footprint index, and their interaction on pollination services within the human‐footprint model.

## 3 Results

### 3.1 Overview of *Caladenia* Pollination Services

The full dataset spanned from 1887 to 2020 (Figure S3). Due to the small sample size prior to 1925, we focused our main analysis on the period 1925–2020 (raw *n* = 10,178) but see Supporting Information for the full dataset analysis. The GAMM identified a significant relationship between pollination service and year (*F* = 3250, *R* ^2^(adj) = 0.745, EDF = 8.939, *p* < 0.001), with an estimated decline in pollination service between 1925 and 2020 of 61%. Segmented regression identified 1977 (±5.6 years) as a significant breakpoint in pollination services. Before 1977, there was no statistically significant relationship observed between year and pollination services (*t* = 1.352, SE = 0.001, df = 79, *p* = 0.180). After 1977, a significant negative relationship was observed, with pollination services declining by 0.76% annually (*t* = −4.609, SE = 0.002, df = 79, *p* < 0.001).

### 3.2 Investigation of Different Pollination Strategies and Ecological Specializations

Mean pollination service differed between species according to pollination strategy (*F*(2, 10,013) = 7.18, *p* < 0.001). Sexually deceptive species exhibited the highest median pollination services, whereas self‐pollinating species had the lowest. Pairwise comparisons revealed a significant difference in pollination services between sexual deception and food deception, whereas other comparisons were not significant (Table S2).

**FIGURE 3:**
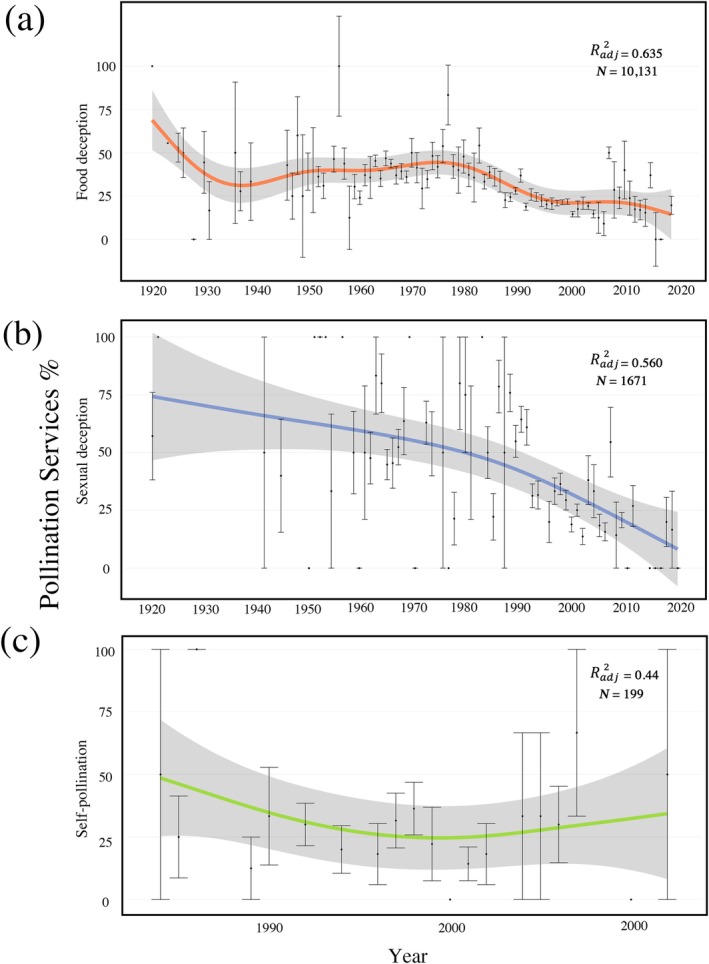
Historical trends of pollination services over time for different pollination strategies. (a) Food‐deceptive species. (b) Sexually deceptive species. (c) Self‐pollinating species. Black points and error bars indicate annual mean pollination services (±SE). Colored lines represent GAMM‐predicted trends for each pollination strategy, with gray shaded areas indicating 95% confidence intervals.

The relationship between pollination services and year differed markedly across pollination strategy (*F*(2, 9988) = 17.15, *p* < 0.001). For food‐deceptive species (Model *n* = 8160 sampled flowers), there was an estimated overall decline in pollination services between 1925 and 2020 of 55.3% (*F* = 2409, EDF = 8.908, *R* ^2^(adj) = 0.727, *p* < 0.001; Figure 3a). A significant breakpoint occurred at 1977 (±6.53 years). Before 1977, pollination services showed no significant relationship with year (*t* = 1.077, 95% CI: −0.001 to 0.005, *R* ^2^ = 0.02, *p* = 0.285). After 1977, pollination services showed a significant relative decline rate of 0.71% per year (*t* = −4.261, 95% CI: −0.010 to −0.004, *R* ^2^ = 0.382, *p* < 0.001).

For sexually deceptive species (Model *n* = 1658 sampled flowers), there was an estimated overall decline in pollination services of 94.1% between 1925 and 2016 (*F* = 302.1, EDF = 8.498, *R* ^2^(adj) = 0.613, *p* < 0.001, Figure 3b). A significant breakpoint occurred at 1989 (±9.46 years). Before 1989, pollination services showed no significant relationship with year (*t*  = −1.202, 95% CI: −0.010 to 0.003, *R* ^2^ = 0.028, *p* = 0.234). After 1989, pollination services showed a significant relative decline rate of 1.74% per year (*t* = −2.892, 95% CI: −0.029 to −0.005, *R* ^2^ = 0.610, *p* < 0.01).

For self‐pollinating species (*n* = 194 sampled flowers), there was a significant relationship between pollination services and year (*F* = 11.30, EDF = 8.021, *R* ^2^(adj) = 0.341, *p* < 0.001; Figure 3c). Segmented regression identified a non‐significant breakpoint in 1994 (±4.2 years), with a non‐significant relationship between pollination services and year before (*t* = −1.537, 95% CI: −0.088 to 0.014, *R*² = 0.221, *p* = 0.144) and after (*t* = 0.855, 95% CI: −0.016 to 0.038, *R*² = 0.087, *p* = 0.405) 1994.

A Kruskal–Wallis test indicated significant differences in median pollination services among species primarily pollinated by either wasps, bees, and flies across the whole period (*χ* ^2^ = 10.706, df = 2, *p* < 0.001). Pairwise Wilcoxon rank‐sum tests with Benjamini‐Hochberg correction indicated marginally higher pollination services for species pollinated primarily by bees compared to wasps (*W* = 4,983,630, *p* = 0.0035), whereas species pollinated by flies and wasps did not differ significantly (*W* = 228,299, *p* = 0.5295). Likewise, pollination services to *Caladenia* pollinated by bees and flies were not significantly different (*W* = 803,049, *p* = 0.5295).

For the period 1925–2020, pollination services were estimated to have declined by 46.34% for species pollinated by wasps, 57.14% for species pollinated by bees, and 38.80% for species pollinated by flies. The relationship between pollination services and year was significant for all groups: wasps (*n* = 3261, EDF = 8.665, *F* = 816.2, *R* ^2^(adj) = 0.676, *p* < 0.001), bees (*n* = 7745, EDF = 8.912, *F* = 2359.6, *R* ^2^(adj) = 0.730, *p* < 0.001), and flies (*n* = 1843, EDF = 8.606, *F* = 499.5, *R* ^2^(adj) = 0.694, *p* < 0.001) (Figure S4).

For species pollinated by wasps, a breakpoint occurred in 1977 (±7.05 years). Before 1983, there was no significant relationship between pollination services and year (*t* = 0.229, 95% CI: −0.005 to 0.006, *p* = 0.821, *R* ^2^ = 0.002). After 1983, pollination services showed a significant annual decline of 0.76% per year (*t* = −6.551, 95% CI: −0.010 to −0.005, *R* ^2^ = 0.537, *p* < 0.001).

For species pollinated by bees, a significant breakpoint was identified at 1977 (±5.11 years). Before this breakpoint, pollination services showed no significant relationship with year (*t* = 1.683, 95% CI: −0.00048 to 0.00523, *R* ^2^ = 0.071, *p* = 0.101). After 1977, pollination services showed a significant annual decline of 0.68% per year (*t* = −5.582, 95% CI: −0.00909 to −0.00442, *R* ^2^ = 0.468, *p* < 0.01).

For the one species pollinated by flies, a breakpoint was identified in 1979 (±7.04 years). Before this breakpoint, pollination services showed no significant change (*t* = 0.010, 95% CI: −0.004 to 0.004, *R* ^2^ < 0.001, *p* = 0.992). After 1948, pollination services showed a significant annual decline of 0.68% per year (*t* = −5.266, 95% CI: −0.009 to −0.004, *R* ^2^ = 0.464, *p* < 0.001).

The decline in pollination service was similar across threat status. For listed species (*n* = 2975), the total decline from 1925 to 2020 was 63.2% (*R* ^2^ = 0.70, edf = 8.87, *F* = 774.1, *p* < 0.001), whereas for unlisted species (*n* = 7202), the total decline was 58.52% (*R* ^2^ = 0.771, edf = 8.87, *F* = 2654, *p* < 0.001) (Figure S5). There was a significant interaction between year and threat status (SE = 0.001, *t* = 3.015, *p* < 0.05) indicating that while the average rates of decline were comparable, the shape or timing of change in pollination differed between listed and unlisted species. Breakpoint analysis indicated high uncertainty around the estimated breakpoints, with overlapping confidence intervals between listed and non‐listed species. In listed species, the breakpoint occurred in 1983 (±18.2 years), where there was no significant relationship between pollination services and year prior to the breakpoint (*t* = −0.87, 95% CI: −0.008 to 0.003, *p* = 0.385) and after there was a significant decline of 0.789% per year (*t* = −2.29, 95% CI: −0.015 to −0.001, *R* ^2^ = 0.229, *p* < 0.05). In unlisted species, the breakpoint occurred 1977 (±4.2 years), before the breakpoint there was a significant positive relationship between pollination services (*t* = 2.70, 95% CI: 0.001–0.006, *p* = 0.008), after 1977, pollination services declined by 0.805% per year (*t* = −5.61, 95% CI: −0.011 to −0.005, *R* ^2^ = 0.402, *p* < 0.001).

### 3.3 Environmental Predictors of Pollination Services

**FIGURE 4:**
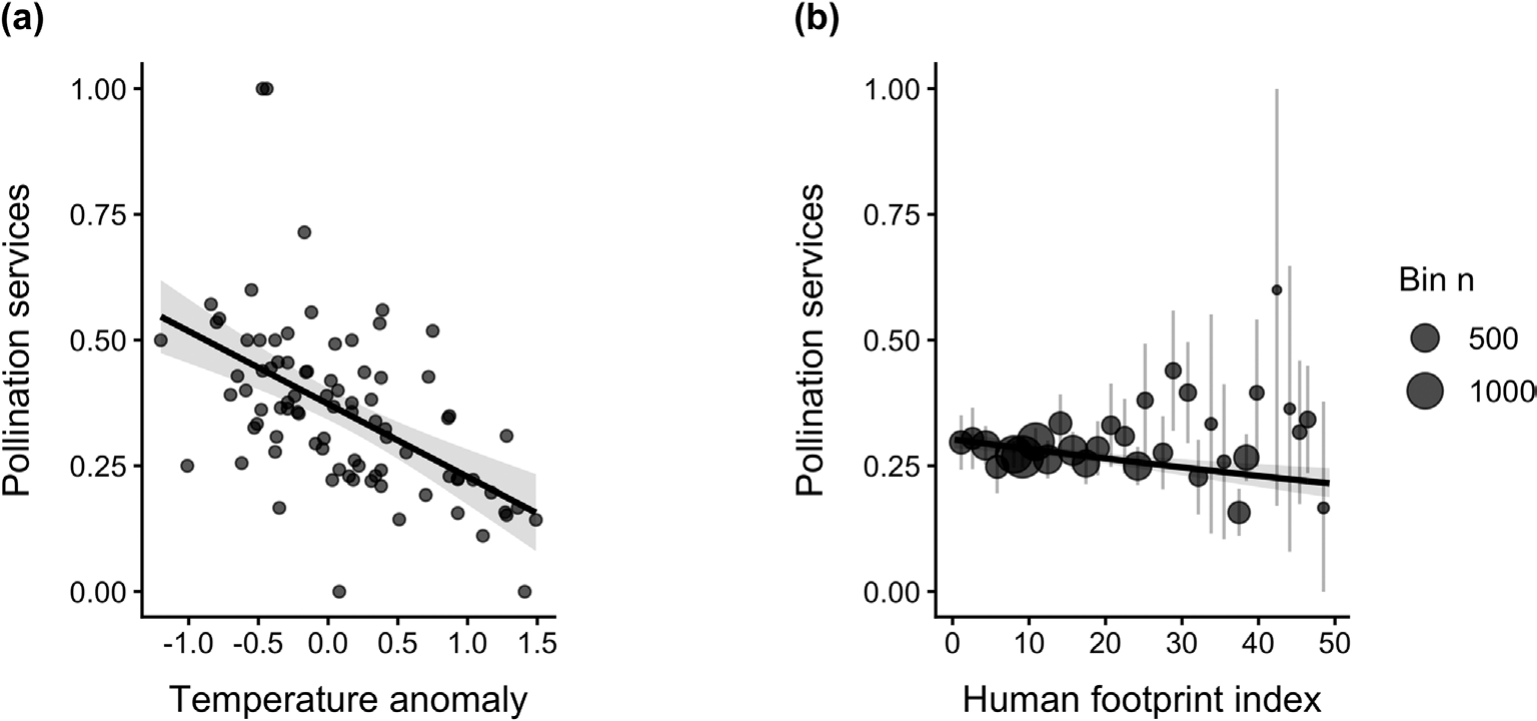
Environmental drivers of pollination services in *Caladenia.* (a) Model‐predicted effect of annual temperature anomaly on pollination services, estimated using a Gaussian generalized linear model. Points show annual mean pollination services; the solid line shows the model prediction with 95% confidence intervals. (b) Partial effect of the human footprint index on pollination services with year statistically controlled, estimated using a binomial generalized linear model. Points represent binned observations (point size proportional to sample size per bin) with binomial confidence intervals. The solid line shows the model‐predicted probability of pollination services with 95% confidence intervals.

Using GLM, we found a significant negative correlation between temperature anomaly and pollination services (*t* = −5.72, SE = 0.025, df = 84, *p* < 0.001; Figure 4a), with pollination services decreasing by approximately 14.5% for every 1°C increase in temperature anomaly.

Pollination services were not correlated with annual rainfall anomalies (*t* = 0.253, SE = 0.001, df = 84, *p* = 0.801). Similarly, pollination services were not correlated with rainfall anomalies for the dominant rainfall period at the collection location, which were summer (*t* = −0.966, SE = 0.001, df = 6, *p* = 0.338) and winter (*t* = 0.916, SE = 0.001, *p* = 0.363).

Human footprint had a significant relationship with pollination services, where areas with higher human footprint had significantly lower pollination services compared to those with a lower human footprint (estimate = 0.0002, *t* = 2.28, *p* = 0.023) (Table S3, Figure S6). Using a GLM with an interaction term, we found a significant interaction between human footprint and year on pollination services, where environments with a lower human footprint experienced a greater decline in pollination services over time (estimate = 9.071e‐05, *t* = 2.26, *p* = 0.024) (Table S3, Figure S6). Human footprint showed a significant negative relationship with pollination services when controlling for temporal trends using a binomial model at the record level (*z* = −3.90, SE = 0.0024, *p* < 0.001; AIC = 10,557; Figure 4b).

## 4 Discussion

Pollination services to *Caladenia* have experienced a decline since the 1970s. This decline was modulated by pollination strategy, with species relying on sexual deception showing the fastest rate of decline, followed by food deception, whereas the self‐pollinating species did not exhibit a decline. Species pollinated by different pollinator taxa experienced similar declines, but our results highlight that more data on pollinator identity are needed to assess whether pollination‐service trends differ among groups. Decline in pollination services to *Caladenia* correlated with increased temperature anomaly, indicating a potential impact of climate change on pollination services. High human footprint index was also associated with a lower mean pollination service for *Caladenia* when considering data across all years. There was also an interaction between time and human footprint, with orchids in less human‐impacted sites experiencing greater declines in pollination service through time. The greater decline in natural sites through time suggests a widespread, overarching environmental driver is responsible for pollination service declines. One possibility is that climate may be the primary driver behind declines in pollination services to orchids, but other environmental factors for which data were not analyzed may also play a role.

Pollination services to the single self‐pollinating species showed no distinct temporal patterns, which may reflect their lower vulnerability to fluctuations in pollinator populations or may simply reflect the limited sample size in this study for this pollination strategy, especially before 1980. There are risks associated with relying on self‐pollination, as self‐pollination can lead to reduced genetic diversity, increasing the risk of genetic drift and inbreeding depression (Goodwillie et al. 2005). Therefore, while self‐pollination may currently provide reproductive insurance, it could reduce adaptive capacity (Jersáková et al. 2006; Patt et al. 1989), which may represent a future risk to these species, particularly under severe ecological pressures such as climate change.

Sexually deceptive *Caladenia* species showed the greatest decline in pollination services compared to other pollination strategies. Sexually deceptive species may be more vulnerable to ecological changes due to their high rates of ecological specialization, including higher rates of one‐to‐one pollinator specialization compared to other orchids (Ackerman et al. 2023), as demonstrated for *Caladenia huegelii* (Phillips et al. 2015). A compounding influence on pollination services to sexually deceptive species, and particularly relevant for threatened species of *Caladenia*, is plant population size. In sexually deceptive orchids, increasing plant density can lead to lower per plant reproductive rates, as pollinators can learn to avoid deceptive flowers (Phillips Reiter et al. 2020). Moreover, sexually deceptive orchids can impact pollinator populations by driving male bias and causing sperm wastage (Brunton Martin et al. 2020, 2021). Male wasps deceived by the flowers ejaculate as if mating with a real female, losing a substantial proportion of their sperm reserves during a single “pseudocopulation,” which in turn reduces the amount of sperm available for subsequent mating with actual females (Gaskett et al. 2008). We were unable to account for plant population size in our study as these data were not consistently recorded with herbarium specimens. A further consideration is the additional layer of specialization caused by reliance on male insects for pollination. It remains unclear how sex differences in sensitivity to disturbance may affect pollination services to these species and this may be a future frontier in pollination ecology.

Food‐deceptive orchids are generally thought to depend on a wider variety of pollinators, although they can still have specific (one‐to‐one) pollinator relationships (Ackerman et al. 2023). Compared to sexually deceptive species, relatively little is known about the pollinators of food‐deceptive *Caladenia*, but they have been shown to have specialized relationships with pollinators or pollinator groups (Phillips and Batley 2020; Phillips et al. 2023; Reiter, Bohman, Batley, and Phillips 2019; Reiter, Bohman, Freestone, et al. 2019). Most sexually deceptive species are known to exploit male thynnine wasps (Phillips et al. 2023). Non‐sexually deceptive species in the genus are associated with a range of taxa including solitary bees, nectar‐feeding pompilid wasps, and nectar‐seeking thynnine wasps, which suggests that shifts in pollination strategy between food and sexually deceptive can occur without changing pollinator family (Phillips et al. 2023).

We found similar overall declines in pollination services in species pollinated by different pollinator taxonomic groups. However, the information on the identity of pollinators for the orchid species in this study were scattered, in many cases scarce and collected via disparate methods. Greater research on pollinator identity in orchids is urgently needed, particularly given the substantial taxonomic gaps and limited ecological knowledge for many species.

Our results suggest broad‐scale drivers are responsible for declines in pollination services since the 1970s. Habitat loss and fragmentation, and introduced species are known to impact pollination services and orchid population persistence (Swarts and Dixon 2009). Here we used the human footprint index, which integrates multiple anthropogenic pressures, including habitat loss, fragmentation and degradation, expansion of crops and pastures, built infrastructure, transportation networks, and increases in invasive species, as a proxy for anthropogenic impact (Venter et al. 2016). We found areas with higher human footprint had lower pollination services overall. However, we also found observed declines in pollination services have occurred at a higher rate in areas that are under lower land‐use intensity. Climate is a possible driver for contemporary declines in pollination services in less‐disturbed regions. In support of this hypothesis, we found a correlative relationship between temperature anomaly and decline in pollination services. Climate change can affect the interaction between plants and their pollinators in multiple ways. Temperature rises can disrupt the synchrony between plant flowering time and insect emergence (Hutchings et al. 2018), but on the other hand, Alquichire‐Rojas et al. (2024) found that warmer temperatures can increase early season pollinator activity and/or duration of foraging. Australia has warmed on average by ~1.5°C since records began in 1910, with a marked rise from the 1950s onward. Since the 1970s, shifting climate drivers like El Niño–Southern Oscillation (ENSO), Indian Ocean Dipole (IOD), and a strengthening Southern Annular Mode (SAM) superimposed over increased mean temperatures have intensified and prolonged extreme heat events nationwide (CSIRO and BOM 2024). These changes are likely to affect pollination services. For example, extreme temperatures in Australia have been shown to affect the activity of solitary bees, decreasing the duration of their active foraging periods, which may decrease pollination services to plants (Rader et al. 2013).

We found no significant correlation between changes in rainfall and pollination services. However, rainfall has been found to correlate with orchid flowering in Australia and elsewhere (Wells et al. 1998; Kéry and Gregg 2004; Jasinge et al. 2018a), and therefore changes in rainfall have the potential to impact pollination services to orchids (Brundrett 2019). Another climate‐linked driver of changes to pollination services is the alteration of fire regimes. High fire frequency, high fire severity, out of season fire, and substrate fires have been identified as threats to Australian ecosystems (Keith et al. 2022), with season of burn having demonstrated effects for orchid growth and survival (Jasinge et al. 2018b; Thomsen et al. 2024). Although a single fire had no effect on the pollination of *Caladenia tessellata* (Phillips et al. 2024), correlation between fire regime and orchid pollination services is an area for further research.

In this study we were unable to test the effects of pesticides directly due to a lack of available data. However, the sharp decline in pollination services detected since the 1970s reflects the uptick in pesticide registration in Australia between the 1960s and 1980s (Brain and Anderson 2019). Together, the effects of climate, land‐use, and pesticide use could collectively undermine pollination services and ultimately impact the reproductive success of plants. Species dependent on specific environmental conditions or on specific pollinator taxa for pollination, which is common among orchids, may be particularly vulnerable to the effects of anthropogenic change, making orchids sentinels for the effects of global change on pollination services (Thomas et al. 2004). In addition to the threats to pollination services, herbaria themselves also face potential risks. Particularly in the United States, many herbaria are experiencing budget cuts or are being closed. Our study reaffirms the vital role of museum and herbarium collections in understanding the effects of global change on biodiversity and the critical contribution they make to society (Bartomeus et al. 2019; Rakosy et al. 2023).

Pollination services play a vital role in the maintenance of terrestrial ecosystems globally and they are under threat from the intensification of human activities. Orchids represent an important part of global plant diversity, and their unique pollination strategies and specialization on pollinator groups make them sentinels of change for other ecologically and economically important plant species. This study documented a major decline in pollination services over the continental landmass of Australia. We identified a tipping point in pollination services starting in the 1970s. Our study indicates that a major overarching driver is responsible for pollination service decline such as rising temperatures and land‐use intensity.

## Author Contributions

Conceptualization: J.M.B., T.M.K., and D.R. Funding Acquisition: J.M.B. and T.K., Methodology: J.M.B., H.Z. and M.C. Data collection: B.S. Formal analysis: B.S. Visualization: B.S. Writing – original draft: B.S. Writing – review and editing: J.M.B., H.Z., T.M.K., and D.R. Supervision: J.M.B. and H.Z.

## Funding

This work was supported by Australian Research Council (DE220100144) and German Academic Exchange Service (57654754).

## Conflicts of Interest

The authors declare no conflicts of interest.

## Supporting information


**Appendix S1:** gcb70793‐sup‐0001‐AppendixS1.docx.

## Data Availability

All raw data, along with the R code used for statistical analyses and figure generation, are publicly available in the Zenodo repository: https://doi.org/10.5281/zenodo.18765424.
