# Supplementary material for: Herbarium Specimens Reveal Long‐Term Decline in Pollination Services Since the 1970s
Source: Glob Chang Biol. 2026 Mar 24;32(3):e70793. doi: 10.1111/gcb.70793 (PMC13010227; doi:10.1111/gcb.70793)
Supplement: Supplementary file 1 — Appendix S1: gcb70793‐sup‐0001‐AppendixS1.docx. [file GCB-32-e70793-s001.docx]

## **Supplementary material**

#### **Critical analysis limitations and strengths**

Bias may occur in this study during the specimen collection stage. Uneven sampling is a well-known problem in herbarium use, as collectors tend to ignore seemingly uninteresting sites in favor of "valuable" sites. For example, sampling rates are higher in nature reserves including remnant vegetation, in otherwise cleared/agricultural landscapes. In addition, larger or taller plants may be selected for sampling within a population. Moreover, specimens collected are often concentrated in easily accessible or frequently visited sites (Lang et al., 2019). For example, for some of the species from eastern NSW covered by this study, there are only a few remaining small habitats, so people take samples repeatedly in the same locations. However, repeated sampling at certain sites also provides an advantage for this study. It provides time-series data for specific regions, enabling analysis of long-term trends in pollination services. This continuous data enhances the study’s ability to assess changes over time, adding robustness to conclusions about regional differences and the impact of human activities on pollination services.

**References**

Lang, P. L., Willems, F. M., Scheepens, J., Burbano, H. A. & Bossdorf, O. 2019. Using herbaria to study global environmental change. *New Phytologist,* 221**,** 110-122.

### Supplementary Figures


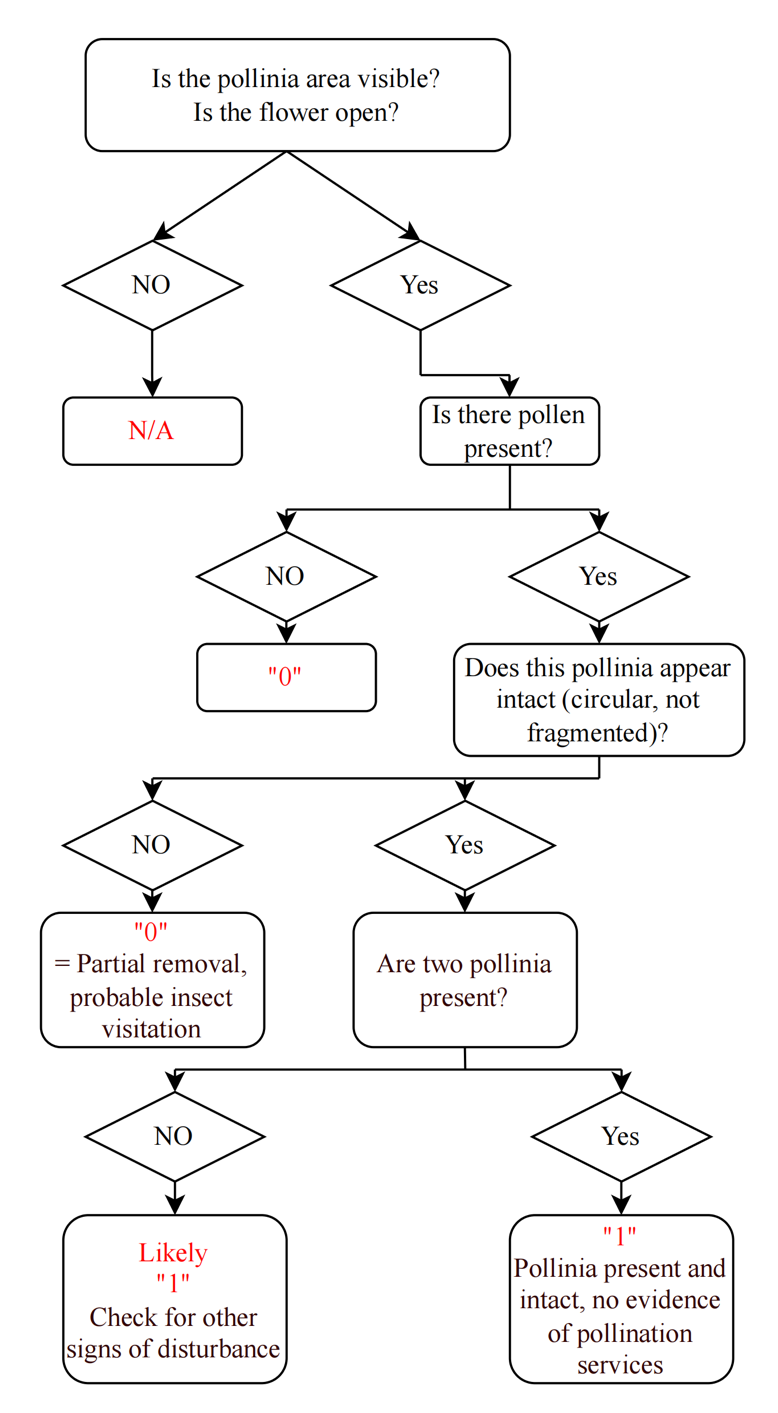


**Figure S1.** **Decision tree for pollinia determination criteria.** The decision tree outlines the stepwise assessment procedure used to evaluate pollinia presence, integrity, and removal in pressed specimens. The workflow begins by verifying floral accessibility and visibility of the anther cap, followed by inspection for the presence of yellow pollinia. Subsequent decision nodes distinguish intact pollinia from partial or complete removal, allowing differentiation between undisturbed flowers, insect-mediated pollinia removal, and potential human-induced damage.


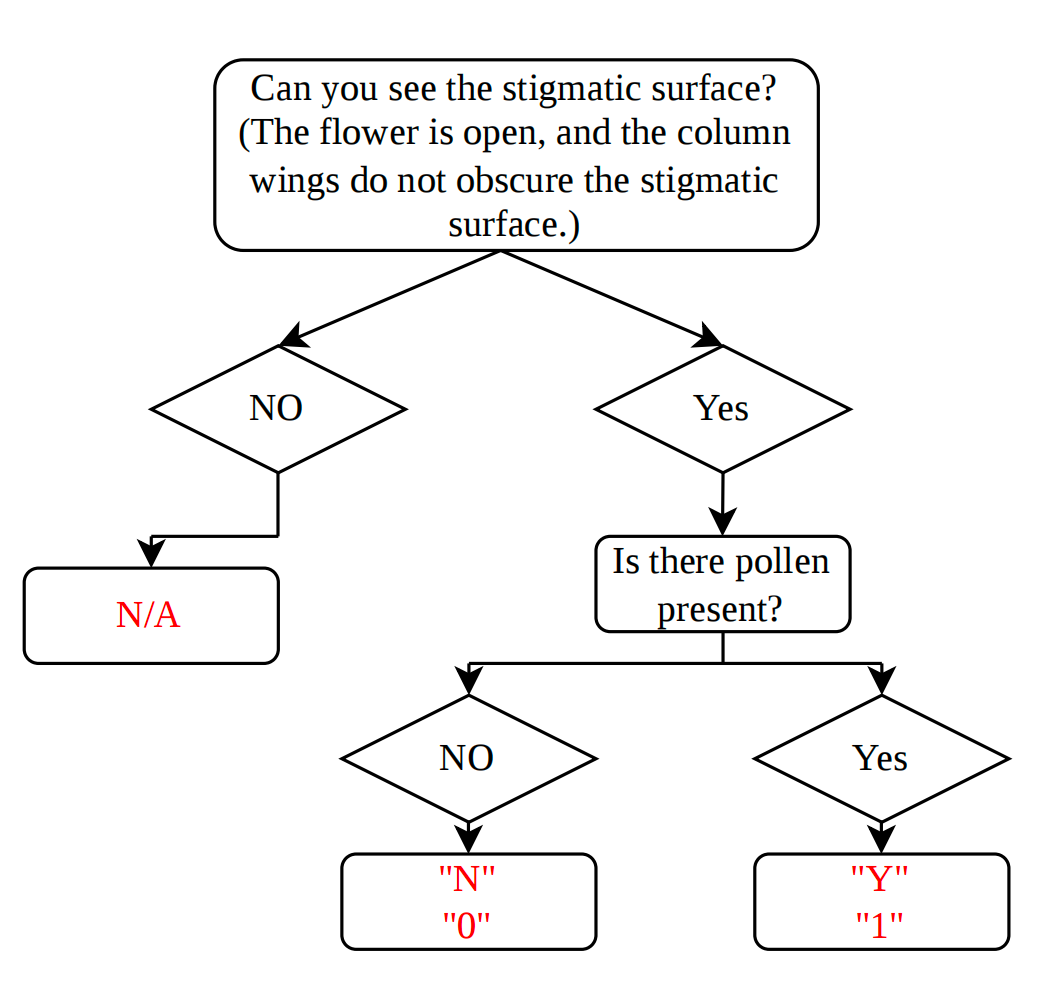


**Figure S2.** **Decision tree for stigma pollen determination criteria.** The assessment begins by checking if the flower is open and the stigmatic surface is visible, followed by examining

whether there is deposited pollen present.


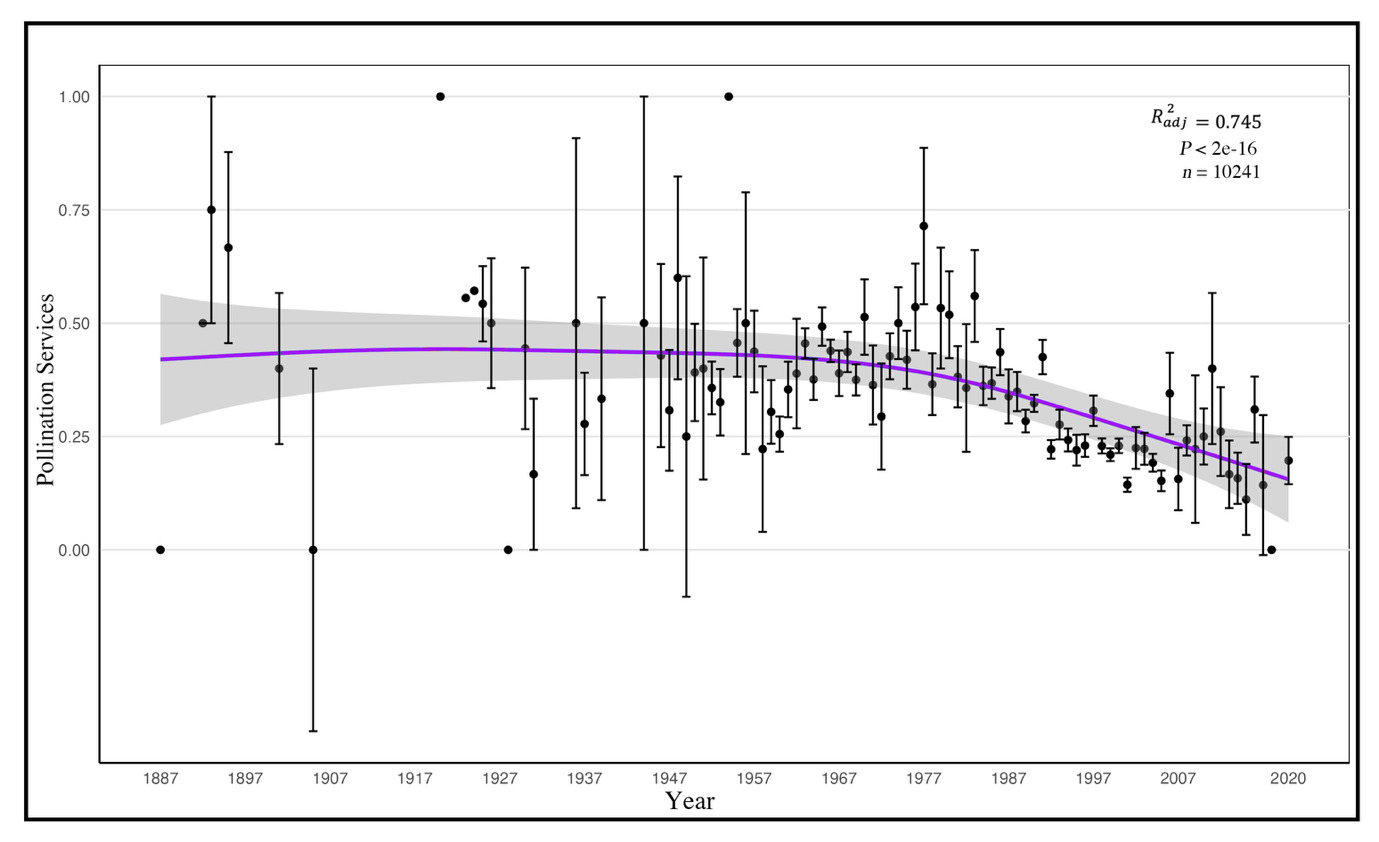


**Figure S3. Historical trends in pollination services to *Caladenia* species (1887–2020).** Temporal trends in mean annual pollination services for *Caladenia* species, expressed as the proportion of specimens receiving pollination services, estimated using generalised additive mixed models (GAMMs) with a Gaussian distribution. Error bars show standard error. The fitted purple line illustrates the modeled trend, and the gray shaded area denotes the 95% confidence interval. Statistical metrics are displayed in the top-right corner.


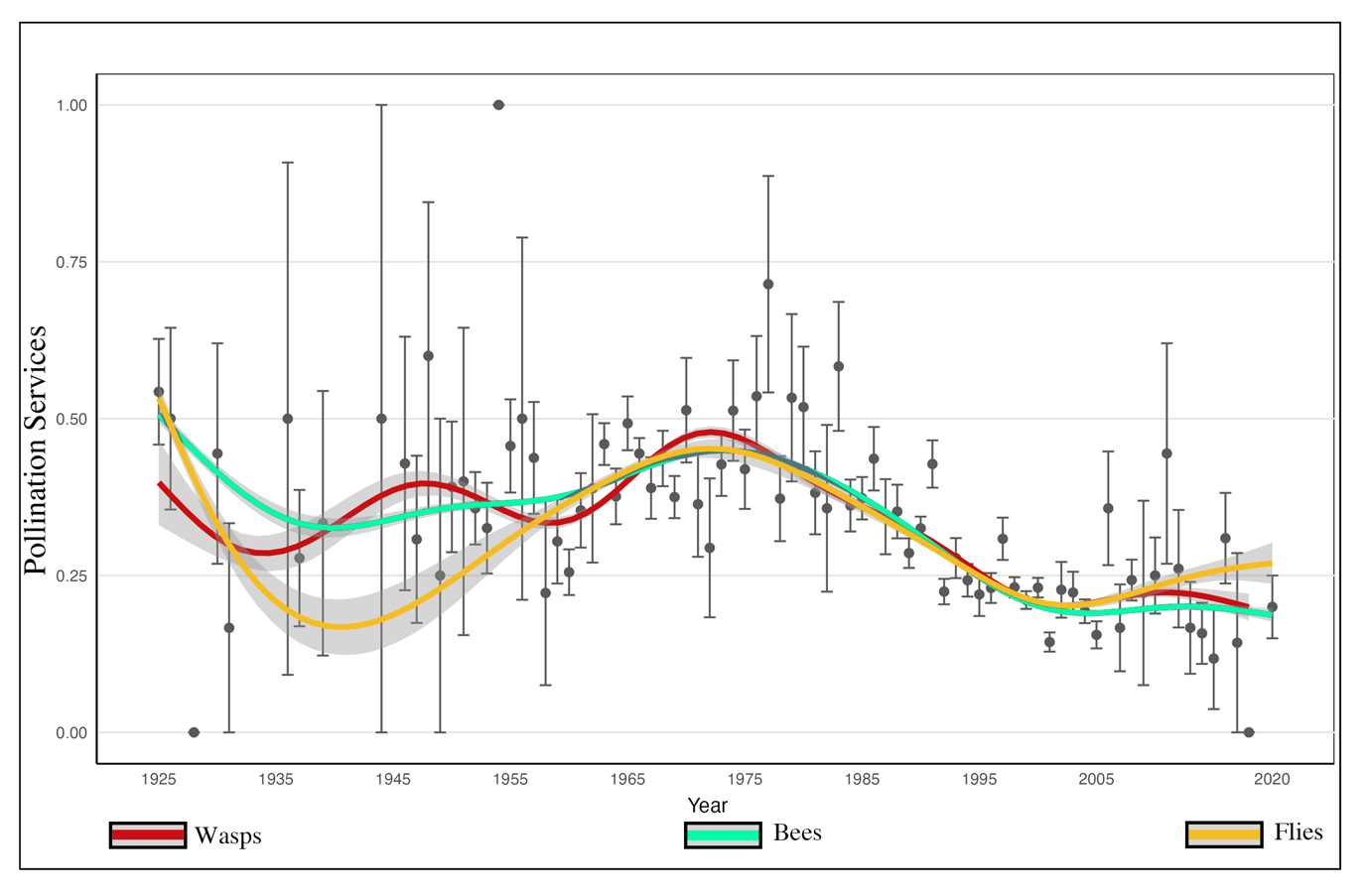


**Figure S4. Temporal trends in pollination services (1925–2020) for *Caladenia* species primarily pollinated by either wasps, bees, or flies.** Data points represent the mean annual pollination service per year as a proportion, with error bars showing the standard error. The colored lines depict the trends predicted by Generalized Additive Mixed Models (GAMMs) with a Gaussian distribution: the red line corresponds to wasps, the green line to bees, and the yellow line to flies. The gray shaded regions around the trendlines indicate the 95% confidence intervals of the fitted models.


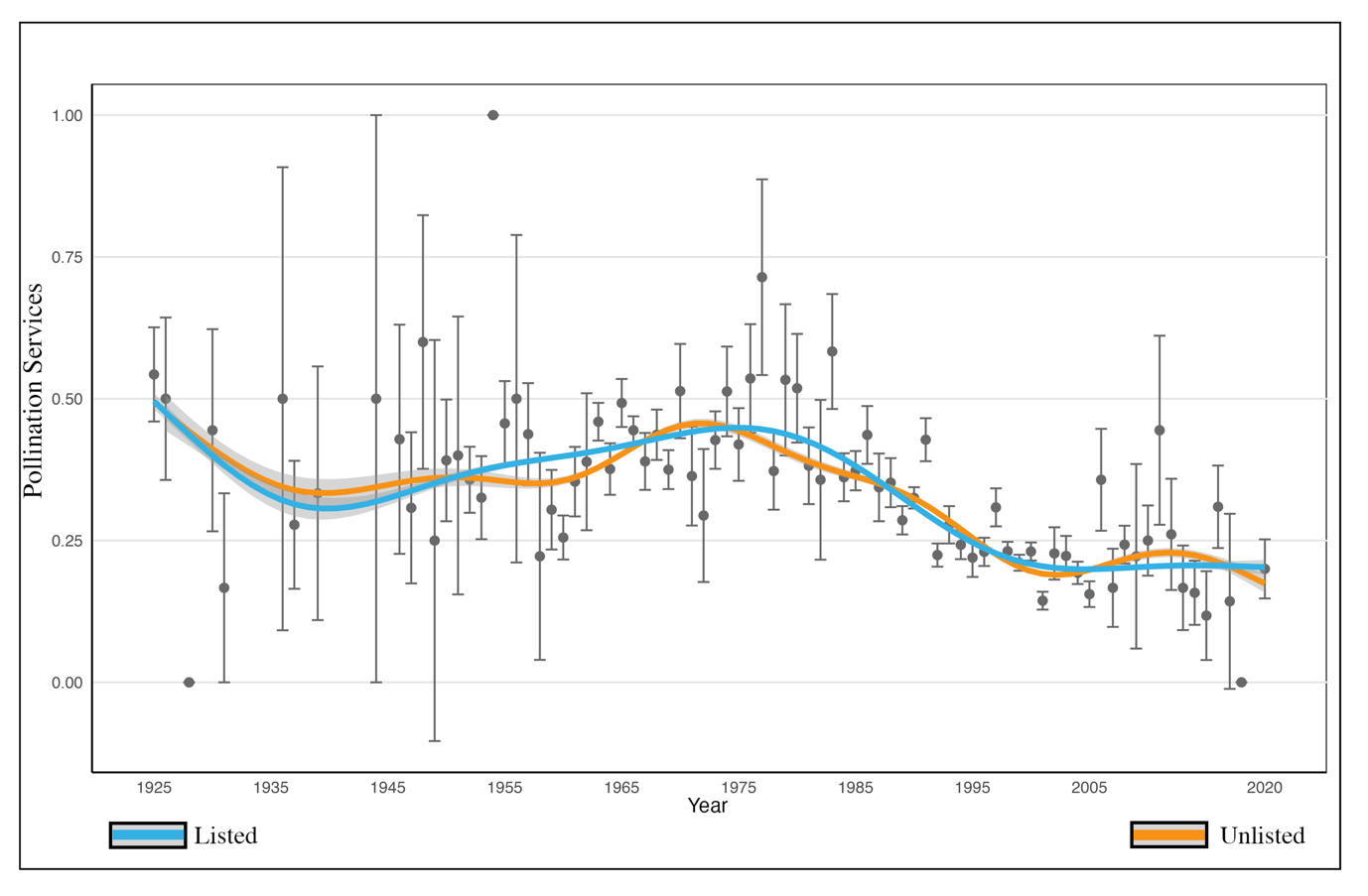


**Figure S5. Temporal trends in pollination services (1925–2020) for *Caladenia* species by threat status.** Data points represent the mean annual pollination service per year as a proportion, with error bars showing the standard error. The colored lines depict the trends predicted by Generalized Additive Mixed Models (GAMMs) with a Gaussian distribution: Blue lines show listed and orange unlisted species. The gray shaded regions around the trendlines indicate the 95% confidence intervals of the fitted models.


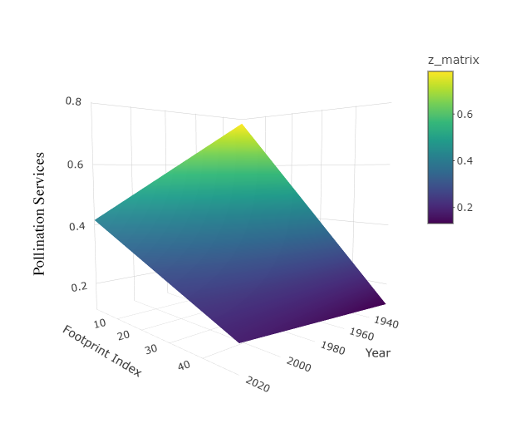


**Figure S6. Three-dimensional interaction plot showing the effect of year and footprint index on predicted pollination services success.** The Z-axis represents predicted pollination services, with higher values indicating more evidence of pollination service. The X-axis represents the footprint index, which quantifies human impact on the environment, and the Y-axis represents the year. The color gradient, ranging from purple (low success) to yellow (high success), visualizes the combined effect of temporal changes and human impact on pollination services.

**References**

AVH. 2026. Australasian Virtual Herbarium: Orchidaceae occurrence records (map view) [Online]. Available: <https://avh.ala.org.au/occurrences/search?taxa=orchidaceae#mapView> [Accessed 8/1/2026].

### Supplementary Table

**Table S1. Detailed information on all study species.** Including sampled numbers, subgenera, pollination syndromes, and pollinator details. The table provides comprehensive details on the studied *Caladenia* and *Glossodia* species, as follows: the sampled number of specimens, their subgenus classification, pollination syndrome, and the corresponding pollinator species or groups. The pollination syndromes include food deception, sexual deception, and self-pollination, highlighting the diversity of reproductive strategies and associated pollinator groups.

| Species Name | Pollination Syndrome | Main pollinator | Orchid Subgenus | Sampled Number  (individual flowers) | Threat  Status | Pollinators and references |
| --- | --- | --- | --- | --- | --- | --- |
| *Caladenia alata* | Self-pollination | NA | *Caladenia* | 223 | Unlisted | Self-pollination (Jones 2021). |
| *C. cardiochila* | Sexual Deception | Wasp | *Calonema* | 119 | Listed | *Phymatothynnus* nr *nitidus* (Phillips et al. 2009); *Phymatothynnus victor* (Bates and Weber 1990). |
| *C. concinna* | Sexual Deception | Wasp | *Calonema* | 138 | Unlisted | Thynnine wasps (*Aelothynnus generosus*; Bower 2001, 2008), *Aelothynnus* sp. (Hayashi 2016 cited by Reiter et al 2019). |
| *C. dilatata* | Sexual Deception | Wasp | *Calonema* | 282 | Listed | *Lophocheilus anilitatis* (Kuiter et al. 2016, p24); *Thynnoides* (given in reference to “most members of the *C. dilatata* complex; Phillips et al. 2009); *Thynnoides* spp. (Stoutamire 1983). |
| *C. filamentosa* | Sexual Deception | Wasp | *Phlebochilus* | 93 | Listed | “?*Chilothynnus* sp.” (Kuiter 2016 p. 52); thynnine wasps (Phillips et al. 2020). |
| *C. parva* | Sexual Deception | Wasp | *Calonema* | 144 | Listed | *Lophocheilus anilitatus* (Kuiter 2016, p. 24); *Lophocheilus anilitatus* (Bower 2015). |
| *C. phaeoclavia* | Sexual Deception | Wasp | *Calonema* | 234 | Unlisted | *Lophocheilus anilitatus* (Bower 2015). |
| *C. reticulata* | Sexual Deception | Wasp | *Calonema* | 100 | Listed | *Phymatothynnus* sp. (Jones 2021); *Phymatothynnus* spp., *Phymatothynnus* nr. *nitidus* 1 (Phillips et al. 2009); *Phymatothynnus nitidus* (Swarts et al. 2014) |
| *C. tensa* | Sexual Deception | Wasp | *Calonema* | 59 | Listed | Thynnine wasps; “*Thynnoides* sp. 1 ? *gracilis*” (Kuiter 2016 p. 26); *Thynnoides gracilis* species, *T. pugionatus* (Bird 2025). |
| *C. tentaculata* | Sexual Deception | Wasp | *Calonema* | 599 | Unlisted | *Thynnoides pugionatus* (Jones 2021); *Thynnoides* sp. 1 (Kuiter 2016 p. 25). |
| *C. alpina* | Food Deception | Bee | *Stegostyla* | 519 | Unlisted | Small native bees (author obs. in Jones 2021). |
| *C. capillata* | Food deception | Multiple/NA | *Phlebochilus* | 168 | Unlisted | Multiple pollinator visitors (Kuiter 2015, 2016). |
| *C. carnea* | Food Deception | Bee | *Caladenia* | 1641 | Unlisted | Native bees (Kuiter 2016, p. 156); *Trigonia* sp. (Adams et al. 1992) *Lasioglossum semipolitum*, *L. clelandi* (Farrington et al. 2009*), Homalictus* sp. (Phillips et al. 2009 [as suggested pollinator]), Sugarbag bees (*Tetragonula carbonaria*; Dyer et al. 2019); native bee (Morrison and Weston 1985). |
| *C. catenata* | Food Deception | Fly | *Caladenia* | 282 | Listed | Hoverfly (*Syrphus* sp.) (Uhlerr 1967); hoverfly (*Melangyna viridiceps*; Kuiter 2016 page 168; Kuiter 2017, p3); *Simosyrphus grandicornis* (Kuiter 2020 p. 56). |
| *C. cucullata* | Food Deception | Multiple/NA | *Stegostyla* | 807 | Listed | Native bees and flower wasps (Phillips et al. 2009). |
| *C. deformis* | Food Deception | Bee | *Pheladenia* | 393 | Unlisted | Small native bees (*Lasioglossum (Chilalictus) hemichalceum* and *L. lanarium*; Kuiter 2016 p 161); small native bees (Jones 2021). |
| *C. fuscata* | Food Deception | Bee | *Caladenia* | 1192 | Unlisted | Native bees (Bates 2011 [re subgenus Elevatae], Phillips et al. 2009); small bee *Lasioglossum* (*Chilalictus*) *hemichalceum* (Kuiter 2016, p. 157). |
| *C. gracilis* | Food Deception | Bee | *Stegostyla* | 226 | Unlisted | Native bees (Phillips et al. 2009); *Exoneura bicolor, Exoneurella lawsonii,* *Lasioglossum globosum, Hylaeus brevior* (Kuiter 2016 p. 158), hoverfly (Kuiter 2016, p. 169). |
| *C. latifolia* | Food Deception | Multiple/NA | *Elevatae* | 562 | Unlisted | Beetles, bee flies (*Comptosia maculosa, Sisyromia*) (Brown et al. 1997), hoverflies (*Malanogyna viridiceps*) (Brown et al. 1997), bees (*Leioproctus* sp.) (Brown et al. 1997), native bees (Bates 2011); small native bees (*Lasioglossum (Chilalictus) globosum*; Kuiter 2016); food deceptive noted by Dixon and Tremblay (2009). |
| *C. moschata* | Food Deception | Bee | *Stegostyla* | 1128 | Listed | *Lasioglossum hemichalceum* (Kuiter 2020, p. 54). |
| *C. patersonii* | Food deception | Multiple/NA | *Calonema* | 73 | Listed | *Phymatothynnus* sp. 6 (Kuiter 2016, p. 48); Tiphiidae, Apidae, Colletidae and Halictidae in Hymenoptera; Calliphoridae, Tachinidae and Syrphidae in Diptera as visitors to the flower, but unsure if pollinators (Stoutamire 1983), food advertising noted by Stoutamire (1983). |
| *C. picta* | Food Deception | NA | *Caladenia* | 140 | Unlisted | Morphology, based on Kuiter (2015, 2016). |
| *C. praecox* | Food Deception | Bee | *Stegostyla* | 325 | Unlisted | Bee (Shrestha et al. 2019) |
| *Glossodia major* | Food Deception | Bee | *Glossodia* | 865 | Unlisted | Native bees (*Lasioglossum hiltacum*, *L. hemichalceum, L. lanarium, Exoneura bicolor*; Kuiter 2016 p. 164); native bees (Jones 2021). |
| *G.minor* | Food Deception | Bee | *Glossodia* | 182 | Unlisted | Small native bees (Jones 2021); *Lasioglossum calophyllae*, *L. hemichalceum*, (Kuiter 2016, p.166). |

**Table S1 References**

Adams P.B., Bartareau, T., Walker, K.L. (1992) Pollination of Australian orchids by *Trigona* (Tetragona) jurine bees (Hymenoptera: Apidae). *Australian Entomological Magazine 19*, 97–101

Bates, R.J., Weber, J.Z. (1990) *Orchids of South Australia.* Government Printer, South Australia.

Bates, R. J. (2011) *South Australia’s Native Orchids*. Native Orchid Society of South Australia Inc.

Bird, A. (2025). *Taxonomy and pollination of the sexually deceptive orchid* Caladenia tensa (Doctoral dissertation, La Trobe).

Bower, C.C. (2001) Determination of the pollinators of sexually deceptive orchids in the subtribes Drakaeinae and Caladeniinae. Technical Report, 1997–2000. Australian Orchid Foundation, Melbourne.

Bower, C.C. (2008) Serendipity and the pollinators reveal a new cryptic spider orchid related to *Caladenia concinna*. *The Orchadian 16, 60-65.*

Bower, C. C. (2015). Pollinator sharing by the sexually deceptive Green-comb Spider Orchids, *Caladenia phaeoclavia, C. parva* and *C. villosissima* (Orchidaceae: Caladeniinae): taxonomic considerations. *Telopea*, *18*, 309-323.

Brown E.M., Burbridge, A.H., Dell, J., Edinger, D., Hopper, S.D., Wills, R.T. (1997) *Pollination in Western Australia: a database of animals visiting flowers. Handbook no. 15*. WA Naturalists’ Club: Perth.

Dixon, K., Tremblay, R. L. (2009). Biology and natural history of *Caladenia. Australian Journal of Botany*, *57*(4), 247-258.

Dyer, A. G., Boyd-Gerny, S., Shrestha, M., Garcia, J. E., van Der Kooi, C. J., & Wong, B. B. (2019). Colour preferences of *Tetragonula carbonaria* Sm. stingless bees for colour morphs of the Australian native orchid *Caladenia carnea*. *Journal of Comparative Physiology A*, *205*(3), 347-361.

Faast, R., Farrington, L., Facelli, J. M., & Austin, A. D. (2009). Bees and white spiders: unravelling the pollination syndrome of *Caladenia rigida* (Orchidaceae). *Australian Journal of Botany, 57(4),* 315-325.

Farrington, L., MacGillivray, P., Faast, R., Austin, A. (2009). Investigating DNA barcoding options for the identification of Caladenia (Orchidaceae) species. *Australian Journal of Botany, 57(4*), 276-286.

Jones (2021) *A complete guide to the native orchids of Australia*. Reed New Holland.

Kuiter, R. (2016) *Orchid Pollinators of Victoria*. Aquatic Photographics,

Kuiter, R. (2017) Pollination of *Caladenia catenata* (Orchidaceae) and its Offspring’s Spatial Pattern of Dispersal. Aquatic Photographics. Short Paper 6

Kuiter, R. (2020). Orchid Pollinators of Victoria. *Victorian Entomologist*, *50*(2). Available online: https://nla.gov.au/nla.obj-2586367430/view

Morrison, D. A., Weston, P. H. (1985). Analysis of morphological variation in a field sample of *Caladenia catenata* (Smith) Druce (Orchidaceae). *Australian Journal of Botany*, *33*(2), 185-195.

Phillips, R. D., Faast, R., Bower, C. C., Brown, G. R., Peakall, R. (2009). Implications of pollination by food and sexual deception for pollinator specificity, fruit set, population genetics and conservation of *Caladenia* (Orchidaceae). *Australian Journal of Botany*, *57*(4), 287-306.

Phillips, R. D., Bohman, B., Brown, G. R., Tomlinson, S., Peakall, R. (2020). A specialised pollination system using nectar‐seeking thynnine wasps in *Caladenia nobilis* (Orchidaceae). *Plant Biology*, *22*(2), 157-166.

Reiter, N., Bohman, B., Freestone, M., Brown, G. R., Phillips, R. D. (2019). Pollination by nectar-foraging thynnine wasps in the endangered *Caladenia arenaria* and *Caladenia concolor* (Orchidaceae). *Australian Journal of Botany*, *67*(7), 490-500.

Stoutamire, W. P. (1983). Wasp-pollinated species of *Caladenia* (Orchidaceae) in south-western Australia. *Australian Journal of Botany*, *31*(4), 383-394.

Shrestha, M., Burd, M., Garcia, J. E., Dorin, A., Dyer, A. G. (2019). Colour evolution within orchids depends on whether the pollinator is a bee or a fly. *Plant Biology, 21(4)*, 745-752.

Swarts, N. D., Clements, M. A., Bower, C. C., Miller, J. T. (2014). Defining conservation units in a complex of morphologically similar, sexually deceptive, highly endangered orchids. *Biological Conservation*, *174*, 55-64.

Ulherr J (1967) A note on the pollination of *Caladenia alba* R.Br. *The Orchadian* 2, 94–195.

**Table S2. Pairwise Comparisons of Pollination Services Among Pollination Syndromes Using Tukey HSD (post-hoc test following one-way ANOVA).** This table summarizes the results of Tukey’s honestly significant difference (HSD) post-hoc comparisons assessing differences in pollination services among three pollination syndromes: self-pollinating, food-deceptive, and sexually deceptive species. The analysis reports the mean difference (diff), 95% confidence intervals (lwr, upr), and Tukey-adjusted p-values for each comparison.

| Comparison | diff | 95%CI (lwr) | 95%CI (upr) | *P-value (adj)* |
| --- | --- | --- | --- | --- |
| Self-pollinating *vs* Food-deceptive | -0.010 | -0.065 | 0.087 | 0.943 |
| Sexually deceptive *vs* Food-deceptive | 0.0456 | 0.017 | 0.074 | <0.001 |
| Sexually deceptive *vs* Self-pollinating | 0.056 | 0.0234 | 0.136 | 0.222 |

**Table S3. Summary of Generalized Linear Model (GLM) Analysis on the Interaction Between Year and Human Footprint Index on Pollination Services.** This table provides the estimated coefficients, standard errors, t-values, and p-values from the GLM model assessing the relationship between year, human footprint index, and their interaction on pollination services.
